# A Comprehensive Review of Bioactive Peptides from Marine Fungi and Their Biological Significance

**DOI:** 10.3390/md17100559

**Published:** 2019-09-29

**Authors:** Fadia S. Youssef, Mohamed L. Ashour, Abdel Nasser B. Singab, Michael Wink

**Affiliations:** 1 Department of Pharmacognosy, Faculty of Pharmacy, Ain-Shams University, Cairo 11566, Egypt; fadiayoussef@pharma.asu.edu.eg (F.S.Y.); ashour@pharma.asu.edu.eg (M.L.A.); 2 Department of Pharmaceutical Sciences, Pharmacy Program, Batterjee Medical College, North Obhur, P.O. Box 6231, Jeddah 21442, Saudi Arabia; 3 Institute of Pharmacy and Molecular Biotechnology, Heidelberg University, Im Neuenheimer Feld 364, 69120 Heidelberg, Germany

**Keywords:** biological activity, chemistry, marine derived fungi, peptides

## Abstract

Fungal marine microorganisms are a valuable source of bioactive natural products. Fungal secondary metabolites mainly comprise alkaloids, terpenoids, peptides, polyketides, steroids, and lactones. Proteins and peptides from marine fungi show minimal human toxicity and less adverse effects comparable to synthetic drugs. This review summarizes the chemistry and the biological activities of peptides that were isolated and structurally elucidated from marine fungi. Relevant fungal genera including *Acremonium*, *Ascotricha*, *Aspergillus*, *Asteromyces*, *Ceratodictyon*, *Clonostachys*, *Emericella*, *Exserohilum*, *Microsporum*, *Metarrhizium*, *Penicillium*, *Scytalidium*, *Simplicillium*, *Stachylidium*, *Talaromyces*, *Trichoderma*, as well as *Zygosporium* were extensively reviewed. About 131 peptides were reported from these 17 genera and their structures were unambiguously determined using 1D and 2D NMR (one and two dimensional nuclear magnetic resonance) techniques in addition to HRMS (high resolution mass spectrometry). Marfey and Mosher reactions were used to confirm the identity of these compounds. About 53% of the isolated peptides exhibited cytotoxic, antimicrobial, and antiviral activity, meanwhile, few of them showed antidiabetic, lipid lowering, and anti-inflammatory activity. However 47% of the isolated peptides showed no activity with respect to the examined biological activity and thus required further in depth biological assessment. In conclusion, when searching for bioactive natural products, it is worth exploring more peptides of fungal origin and assessing their biological activities.

## 1. Introduction

Hundreds of secondary metabolites obtained from marine fungal strains revealed potent pharmacological and biological activity [1]. These mainly comprise alkaloids, terpenoids, and peptides, in addition to polyketides, steroids, and lactones. Relevant bioactivities include antibacterial, anticancer, anti-inflammatory, and antiviral activity [2]. The great diversity in the structure and function of the metabolites derived from marine organisms is mainly attributed to the extensive variation in the chemical and physical conditions of the environment in which the marine organisms survive [3]. As many marine organisms are sessile, they need chemical protection against predators and pathogens.

Marine microorganisms represented by fungi and bacteria, but also marine invertebrates, are regarded as a valuable source of bioactive compounds. Marine microorganisms have the advantage that they can be cultured and thus offer high reproducibility and an everlasting source of natural products [4]. A considerable number of drugs already exist in the market that are of fungal or bacterial origin such as griseofulvin, fucidin, penicillins, and many ergot alkaloids containing products. However, the number of marine fungal metabolites is still quite small [5].

Besides, a number of marine fungal metabolites are characterized by appropriate oral-bioavailability and suitable physico-chemical properties that meet the criteria of formulating effective pharmaceuticals [1]. Furthermore, most fungal proteins and peptides show minimal human toxicity and less adverse effects compared to drugs of synthetic origin [6].

Regarding the history of bioactive peptide isolation from marine organisms, it is noteworthy to highlight that during the past half century hundreds of peptide antibiotics have been explored. They are classified as synthetic peptides (non-ribosomal) and natural (ribosomal) ones. The former are represented by glycopeptides, gramicidins, bacitracins, and polymyxins as well and they are extensively modified and produced mainly by bacterial strains, meanwhile, the latter are equally produced by all species comprising fungi and bacteria and considered as a primary line of defence elicited by these organisms [7].

However, naturally occurring peptides obtained from marine sources possess strongly modified structures either in its backbone or side chain compared to peptides of human origin that undoubtedly do due to the environmental conditions in which they live. This ultimately makes them good candidates for drug design offering great stability from enzymatic degradation as well as thermal conditions. Most of these peptides are taken from ascidians, sponges, as well as mollusks. Besides, wide array of bioactive peptides are produced as the result of the association that exists between microorganisms and the marine organisms. This symbiosis, in turn, generated biochemical pathways in both the marine organisms and its associated microorganisms with concomitant production of many beneficial pharmaceuticals that are of natural origin [8]. 

Peptides are generally isolated from the fermented fungal biomass culture media via extraction of the culture media using appropriate solvents, most commonly ethyl acetate, and then subjected to evaporation till dryness under vacuum at 40 °C to a semi solid residue. The obtained residue was subjected to a series of chromatographic fractionation using traditional stationary phases such as silica gel and sephadex as well, with concomitant purification using high performance liquid chromatography (HPLC) to fully purify and isolate individual peptides in their single forms [9]. 

Structural elucidation and characterization of the isolated peptides are unambiguously determined by spectroscopic analysis comprising 1D and 2D NMR techniques, in addition to mass spectrometry. Their absolute configurations were further ascertained by Marfey’s methods, chemical structural modification, as well as Mosher’s reaction [10]. In addition, ESIMS (Electrospray Ionization Mass Spectrometry) analyses of the free amino acids obtained by acid hydrolysis, as well as HPLC analysis of Marfey products prepared from the acid hydrolysate are also used for determination of peptides structures. However, the peptide amino acid sequence can be also unambiguously determined by MSn ion-trap ESI mass spectrometry [11].

Thus, in this review we comprehensively explore the chemistry and the biological activity of the peptides that were isolated and structurally elucidated from marine fungi of the genera *Acremonium*, *Ascotricha*, *Aspergillus*, *Asteromyces*, *Ceratodictyon*, *Clonostachys*, *Emericella*, *Exserohilum*, *Microsporum*, *Metarrhizium*, *Penicillium*, *Scytalidium*, *Simplicillium*, *Stachylidium*, *Talaromyces*, *Trichoderma*, and *Zygosporium.* Collection of data was done until July 2019 and classification was performed based upon the fungal genera, which are arranged in an alphabetical order. Additionally, a table summarizing the bioactive peptides isolated from marine associated fungi, their sources, and biological activities was added for better illustration of the collected data. 

## 2. Bioactive Peptides in Particular Fungal Genera

### 2.1. Acremonium 

**Figure 1:**
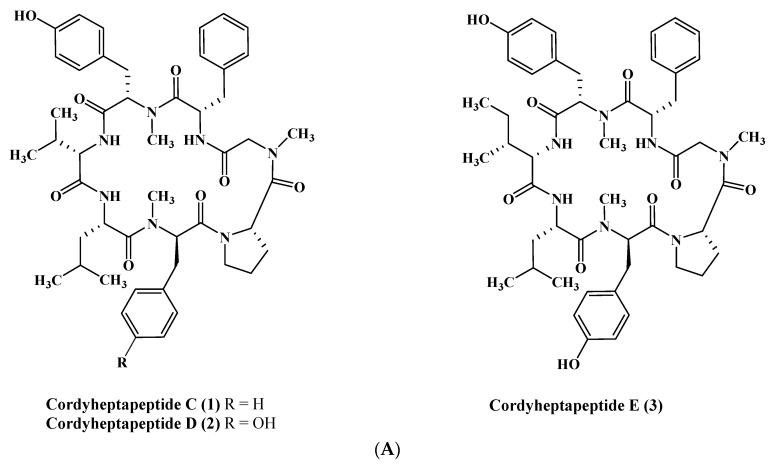

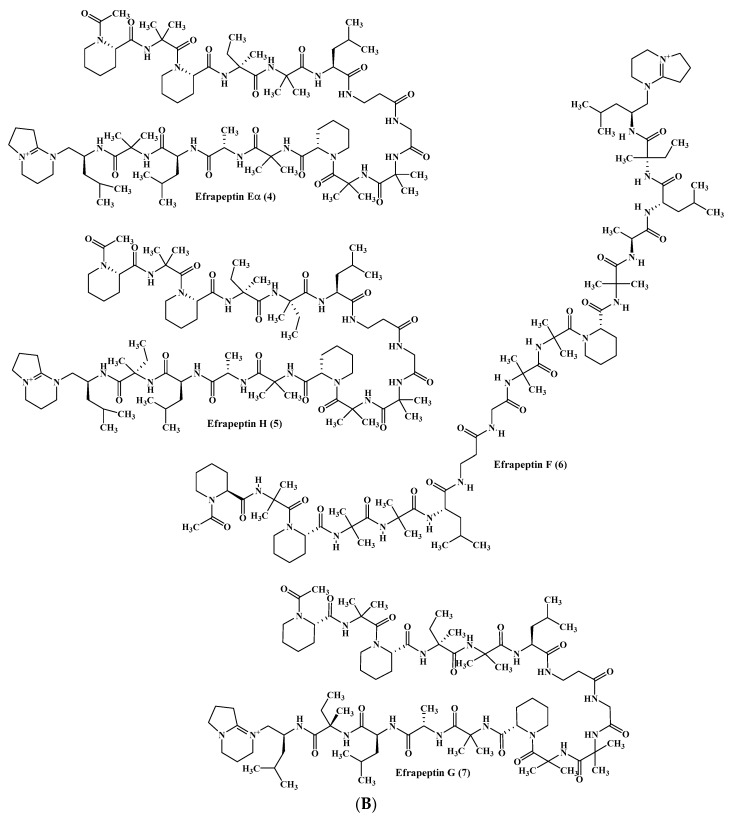

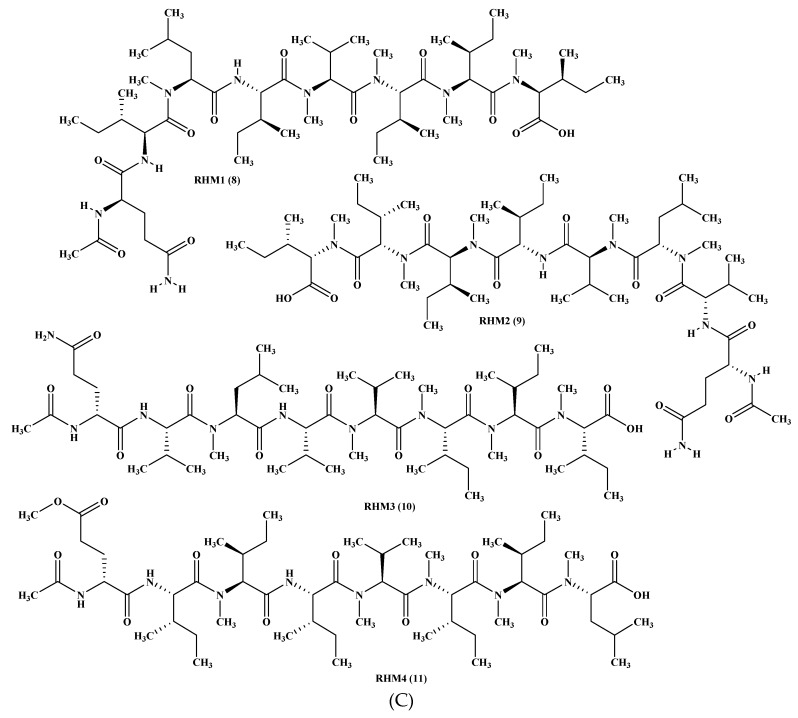
(**A**) Cordyheptapeptides isolated from *Acremonium* species. (**B**) Efrapeptins isolated from *Acremonium* species. (**C**) RHM family isolated from *Acremonium* species.

From a fermenter-culture of *Acremonium persicinum*, three new compounds of cycloheptapeptide skeleton were isolated, namely cordyheptapeptides C–E (**1**–**3**). Only compounds (**1**) and (**3**) showed a substantial cytotoxicity versus MCF-7, SF-268, and NCI-H460 cancer cells displaying IC_50_ values between 2.5 and 12.1 μM [12]. Moreover, two new linear pentadecapeptides (efrapeptins Eα (**4**) and H (**5**)) in addition to known efrapeptin F–G (**6**–**7**) were isolated from an atypical *Acremonium* species. Additionally, RHM1 (**8**), RHM2 (**9**), RHM3 (**10**), and RHM4 (**11**), N-methylated linear octapeptides, were also isolated and structurally elucidated using different spectroscopic techniques from the same species (Figure 1A–C). Promising cytotoxic activity was exhibited by efrapeptins Eα (**4**), F (**6**), and G (**7**) versus H125 cells with an IC_50_ value about 1.3 nM; the latter also showed potent cytotoxic activity versus murine L1210 cells as well as versus HCT-116 with IC_50_ values equal to 0.002 nM. Pronounced antibacterial activity was observed for RHM1 (**8**) as well as for efrapeptin G (**7**) against *Staphylococcus epidermidis* with MIC (Minimum Inhibitory Concentration) values equal to 0.015 and 0.049 μM, respectively [13,14].

### 2.2. Ascotricha 

**Figure 2:**
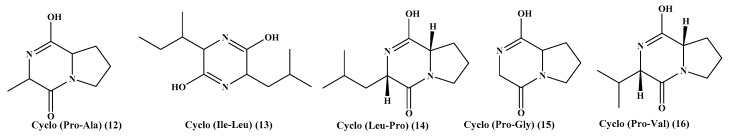
Peptides isolated from *Ascotricha* species.

In depth chemical investigation of the marine fungus *Ascotricha* using different chromatographic techniques resulted in the isolation of 10 compounds that are fully elucidated using various spectroscopic techniques in addition to their physicochemical characteristics. Among them are five peptides namely cyclo (Pro-Ala) (**12**), cyclo (Ile-Leu) (**13**), cyclo (Leu-Pro) (**14**), cyclo (Pro-Gly) (**15**), and cyclo (Pro-Val) (**16**) (Figure 2) that were first to be isolated from genus *Ascotricha* [15].

### 2.3. Aspergillus 

**Figure 3:**
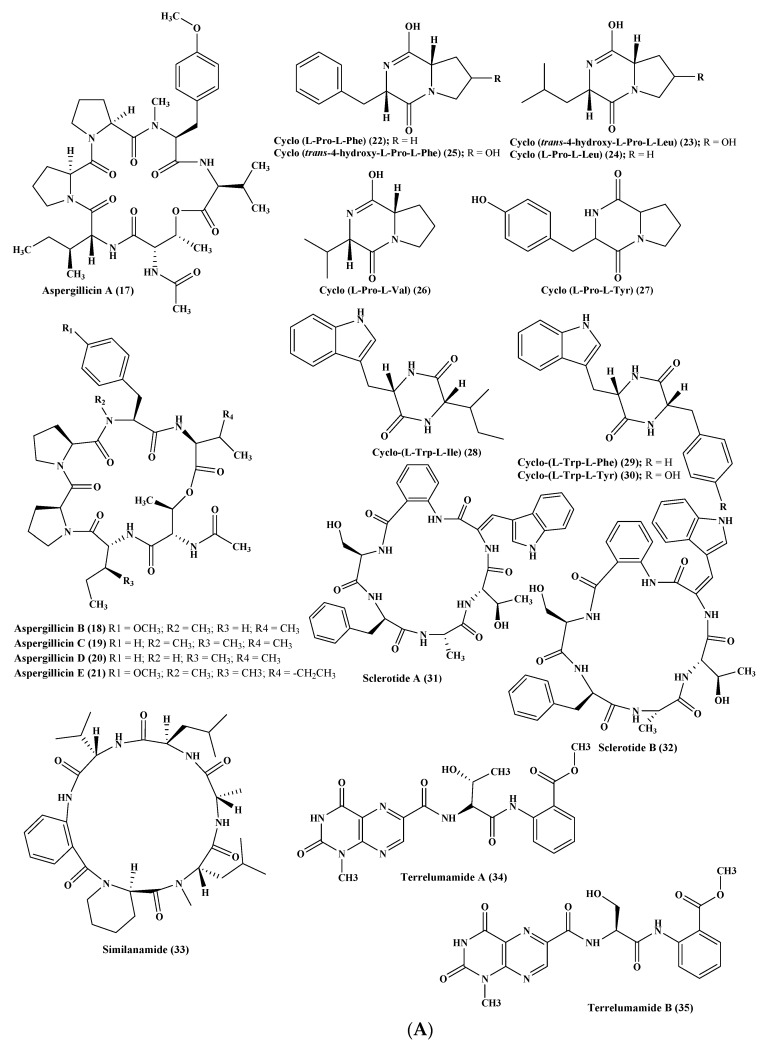

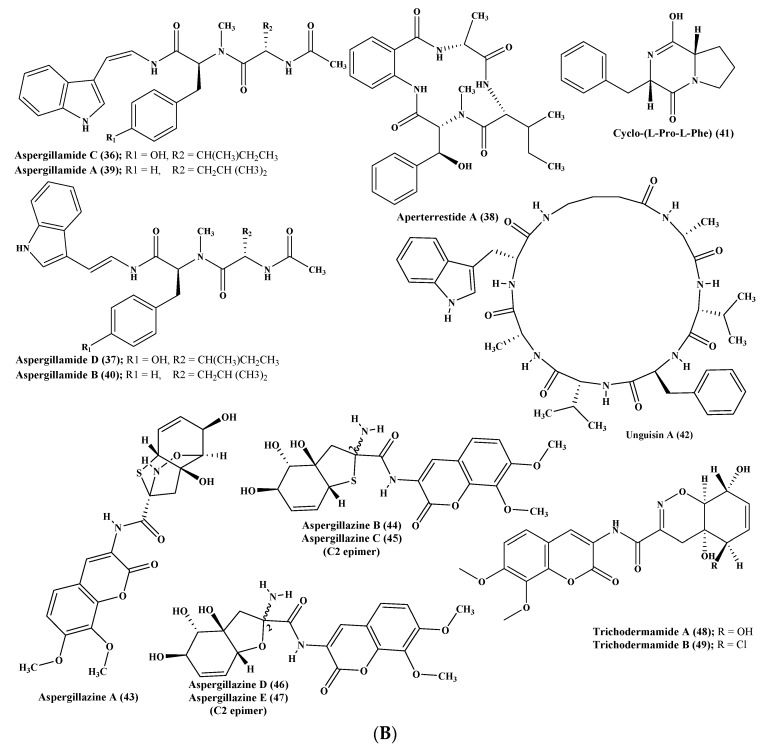

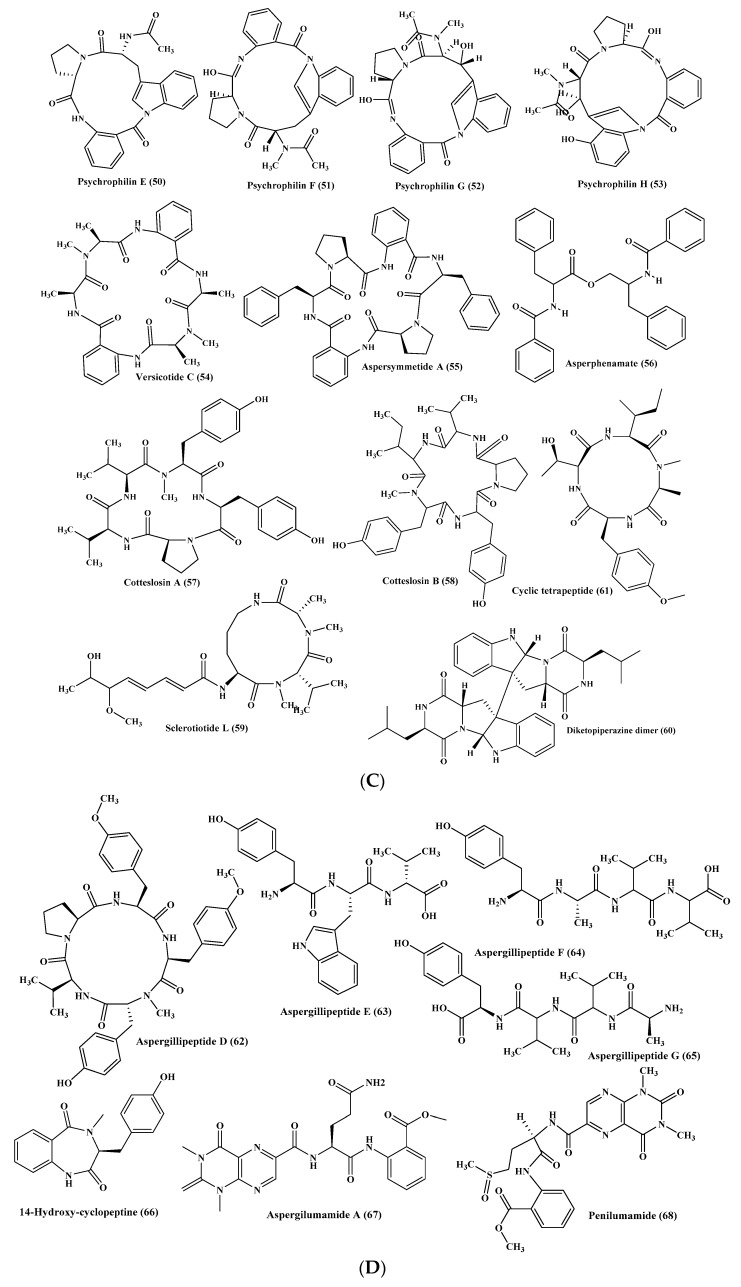
(**A**) Peptides isolated from Aspergillus carneus, A. niger, A. sclerotiorum, and A. terreus. (**B**) Peptides isolated from A. terreus, A. unguis, and A. unilateralis. (**C**) Peptides isolated from A. versicolor and A. violaceofuscus. (**D**) Peptides isolated from miscellaneous *Aspergillus* species.

Five new depsipeptides, aspergillicins A–E (**17**–**21**), were isolated from *Aspergillus carneus* upon comprehensive chemical investigation of a fermenter culture that showed no anti-parasitic activity versus *Haemonchus contortus* in contrast to modest cytotoxic activity with LD_99_ values ranging between 0.03–0.07 μM [11]. Regarding *A. niger*, which was obtained from sediments from the northeast coast of Brazil, then cultured in various growth media, many cyclopeptides were obtained comprising cyclo (l-Pro-l-Phe) (**22**), cyclo (*trans*-4-hydroxy-l-Pro-l-Leu) (**23**), cyclo (l-Pro-l-Leu) (**24**), cyclo (*trans*-4-hydroxy-l-Pro-l-Phe) (**25**), cyclo (l-Pro-l-Val) (**26**), as well as cyclo (l-Pro-l-Tyr) (**27**). The isolated cyclopeptides exhibited no cytotoxic effect against HCT-116 cell line [16]. In addition, other three cyclopeptides namely cyclo-(l-Trp-l-Ile) (**28**), cyclo-(l-Trp-l-Phe) (**29**), and cyclo-(l-Trp-l-Tyr) (**30**) were also reported. The first two cyclopeptides were considered to be plant growth regulators whereas the latter effectively stimulates the differentiation of HT-29 cancer cells. However, neither compounds (**28**–**30**) showed pronounced antimicrobial activity when tested against either *Staphylococcus aureus*, *Escherichia coli*, *A. niger*, or *Candida albicans* [17]. In addition, two novel hexapeptides were isolated and structurally elucidated from the fungus *A. sclerotiorum*. These hexapeptides were sclerotide A (**31**) and sclerotide B (**32**) which showed modest antifungal activity versus *C. albicans* showing MIC values of 7.0 and 3.5 µM, respectively. Additionally, sclerotide B (**32**) revealed selective activity versus *Pseudomonas aeruginosa* with MIC equals to 35.3 µM, meanwhile, it exerted weak cytotoxic activity versus HL-60 cell line with IC_50_ equal to 56.1 µM [18] (Figure 3A).

One more new cyclic hexapeptide, similanamide (**33**), was isolated from a sponge derived fungus, *A. similanensis*, which displayed weak cytotoxic activity versus MCF-7, A373, and NCI-H460 cancer cells with GI_50_ values equal to 0.2, 0.18, and 0.18 µM, respectively, with no antibacterial activity as the (MIC value was found to be greater 256 µg/mL (0.4 µM)) [19]. Moreover, two new lumazine peptides namely terrelumamides A (**34**) and B (**35**), in addition to two new isomeric modified tripeptides, aspergillamides C (**36**) and D (**37**), and a cyclic tetrapeptide asperterrestide A (**38**) were isolated and unambiguously elucidated from *A. terreus*, which is a marine fungus. Compounds (**34**) and (**35**) displayed a promising improvement in insulin sensitivity as determined by the utilization of mesenchymal cells of a bone marrow origin obtained from human (hBM-MSCs) adopting an adipogenesis model. They exerted their action via increasing the formation of adiponectin during the process of adipogenesis in hBM-MSCs with EC50 values equal to 37.1 and 91.9 μM, for terrelumamides A (**34**) and B (**35**), respectively. It is noteworthy to highlight that the maximum elevation in adiponectin levels via terrelumamide A (**34**) induction was 56.9% comparable to that generated by glibenclamide (the standard anti-hyperglycaemic agent). Meanwhile compound (**38)** showed a potent cytotoxic effect versus U937 and MOLT4 human cancer cell lines with IC50 equal to 6.4 and 6.2 μM, respectively, in addition to a pronounced inhibitory potential versus an influenza virus of H1N1, as well as H3N2 strains with IC50 equal to 15 and 8.1 μM, respectively, that relied upon the presence of a 3-OH-N-CH3-Phe moiety that is rare in nature [20,21,22]. Besides, other known peptides were isolated from the latter *Aspergillus* species, which are aspergillamide A **(39)** and B (**40**) and cyclo-(l-Pro-l-Phe) (**41**) [21]. *A. unguis* is another marine fungus from which unguisin A (**42**), which is a cyclic peptide, was isolated [23]. In addition, *A. unilateralis* revealed a complex array of secondary metabolites comprising the heterocyclic dipeptides, aspergillazines A–E (**43**–**47**), which are dipeptides, in addition to trichodermamide A and B (**48**–**49**) [24] (Figure 3B).

Furthermore, four new compounds, psychrophilins E–H (**50**–**53**) were isolated from *A. versicolor*, and they are cyclic peptides characterized by the presence of a rare linkage of amide type between anthranilic acid through its carboxylic acid and the indole ring via its nitrogen. Additionally, a new cyclic hexapeptide versicotide C (**54**) was isolated from the same fungus. All the isolated peptides showed no cytotoxicity, however compound (**52**) exhibited a pronounced lipid-reducing activity at approximately 10 μM as determined by oil red O staining assay [25]. Moreover, secondary metabolites isolated from coral derived *A. versicolor* revealed the presence of a new centrosymmetric cyclohexapeptide, namely aspersymmetide A (**55**), in addition to asperphenamate (**56**) which is a known peptide. Noteworthy to mention is that compound (**55**) was the first centrosymmetric cyclohexapeptide to be isolated from fungi, however it showed weak cytotoxicity versus NCI-H292, as well as A431 cell lines, at 10 µM [26]. Besides, two new cyclopentapeptides, namely cotteslosins A (**57)** and B (**58**), were isolated from the same fungus, *A. versicolor.* Cotteslosin A (**57**) displayed weak cytotoxicity versus human melanoma (MM418c5), prostate (DU145), and breast (T47D) cells with EC_50_ values of 0.1, 0.14, and 0.15 µM, respectively [27]. Concerning *A. violaceofuscus*, a sponge associated fungus, three peptides, which are cyclic, were isolated from its ethyl acetate extract and they were first to be isolated in nature. These cyclic peptides are termed, aspochracin-type cyclic tripeptide sclerotiotide L (**59**), diketopiperazine dimer (**60**), in addition to a cyclic tetrapeptide (**61**) where compounds (**60**–**61**) showed potent anti-inflammatory activity against IL-10 expression of the (Lipopolysaccharide) LPS-induced THP-1 cells (acute monocytic leukemia cell) as evidenced by its inhibition rates which were estimated to be 84.3 and 78.1%, respectively at 10 µM [28] (Figure 3C).

Various peptides were isolated from miscellaneous *Aspergillus* species exemplified by psychrophilin E (**50**), a cyclic tripeptide isolated from two algae associated *Aspergillus* sp whose selectivity inhibited the proliferation of HCT116 (colon) cell line with an IC_50_ value of 28.5 μM comparable to the standard drug cisplatin that showed IC_50_ of 33.4 μM [29]. Additionally, many *Aspergillus* sp. derived peptides displayed antiviral activity such as aspergillipeptides D–G (**62**–**65**), in which aspergillipeptides D (**62**) and E (**63**) displayed a potent antiviral effect versus herpes simplex virus type 1 (HSV-1) displaying IC_50_ of 9.5 and 19.8 µM, respectively, showing no cytotoxic effect at these concentrations versus Vero cell line, meanwhile aspergillipeptide D (**62**) also exhibited activity versus acyclovir resistant clinical isolates of HSV-1 [30]. Regarding the inhibition of nitric oxide production, it was found that the novel cyclic dipeptide isolated from an *Aspergillus* sp., 14-hydroxy-cyclopeptine (**66**), effectively inhibits nitric oxide production displaying IC_50_ of 0.14 μM in recombinant mouse interferon-γ-activated macrophage-like cell line as well as in a lipopolysaccharide without cytotoxic effect at 0.34 μM [31]. Many other peptides were also isolated from *Aspergillus* sp. derived from *Bruguiera sexangula* var. *rhynchopetala* as aspergilumamide A (**67**) and penilumamide (**68**) [32] (Figure 3D).

### 2.4. Asteromyces 

**Figure 4:**
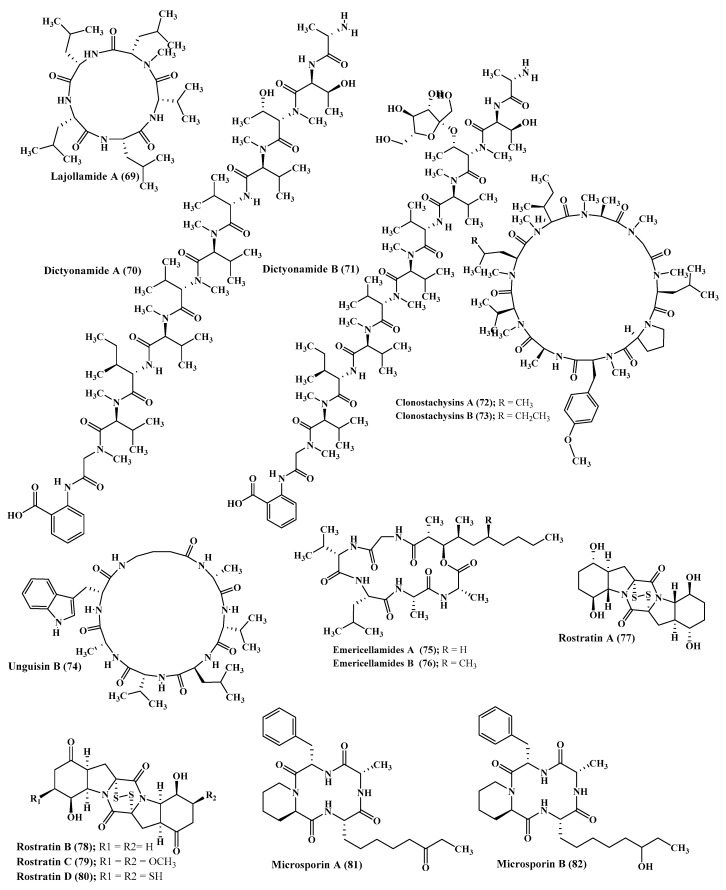
Peptides isolated from Asteromyces, Ceratodictyon, Clonostachys, Emericella, Exserohilum, and Microsporum species.

A new pentapeptide compound, lajollamide A (**69**) was isolated from marine *Asteromyces cruciatus*. This newly isolated peptide showed a weak antimicrobial effect versus Gram-positive bacteria at 100 μM showing 61% and 30% inhibition in bacterial growth for *Bacillus subtilis* and *Staphylococcus epidermidis*, respectively, whereas it showed no inhibition of acetyl cholinesterase activity [33] (Figure 4).

### 2.5. Ceratodictyon 

In depth chemical investigation of the red algae associated fungus, *Ceratodictyon spongiosum*, resulted in the isolation of two new peptides which are dictyonamides A and B (**70**–**71**). The former displayed a pronounced inhibition on cyclin-dependent kinase 4 with IC_50_ value equals to 0.01 μM however the latter showed no inhibition [34] (Figure 4).

### 2.6. Clonostachys 

Clonostachysins A and B (**72**–**73**) are two new cyclic peptides isolated from *Clonostachys rogersoniana* that revealed potent inhibitory activity on a dinoflagellate *Prorocentrum micans* at a concentration of 30 µM but revealed no activity to either bacteria or microalgae [35] (Figure 4).

### 2.7. Emericella 

Unguisins A (**42**) and B (**74**) are two cyclic heptapeptides isolated from the fungus *Emericella unguis* [36]. Besides, emericellamides A and B (**75**–**76**) were also isolated from certain *Emericella* sp. that is co-cultured from *Salinispora arenicola*. Both compounds (**42**) and (**76**) showed encouraging antimicrobial activity against MRSA (methicillin resistant *Staphylococcus aureus* strains) displaying MIC values equal to 3.8 and 6.0 µM, respectively [37] (Figure 4).

### 2.8. Exserohilum 

Rostratins A–D (**77**–**80**) were first to be isolated from the broth of *Exserohilum rostratum*, a marine associated fungus that showed pronounced cytotoxic activity versus (HCT-116) the human colon carcinoma showing 20, 4.48, 1.6, and 34 nM as IC_50_ values, respectively [38] (Figure 4).

### 2.9. Microsporum 

The marine associated fungus, *Microsporum* cf. *gypseum*, yielded two new peptides microsporins, A and B (**81–82**), which effectively inhibit histone deacetylase with IC_50_ values equal to 0.14 and 0.55 µM against (Histone Deacetylases) HDACs and HDAC8, respectively, in addition to displaying a considerable cytotoxic effect versus human colon adenocarcinoma (HCT-116) with IC_50_ values of 1.17 and 16.5 nM, respectively. Additionally, microsporin A (**81**) displayed notable activity against the 60 cancer cell panel of the National Cancer Institute with mean IC_50_ of 2.7 µM [39] (Figure 4).

### 2.10. Metarrhizium

**Figure 5:**
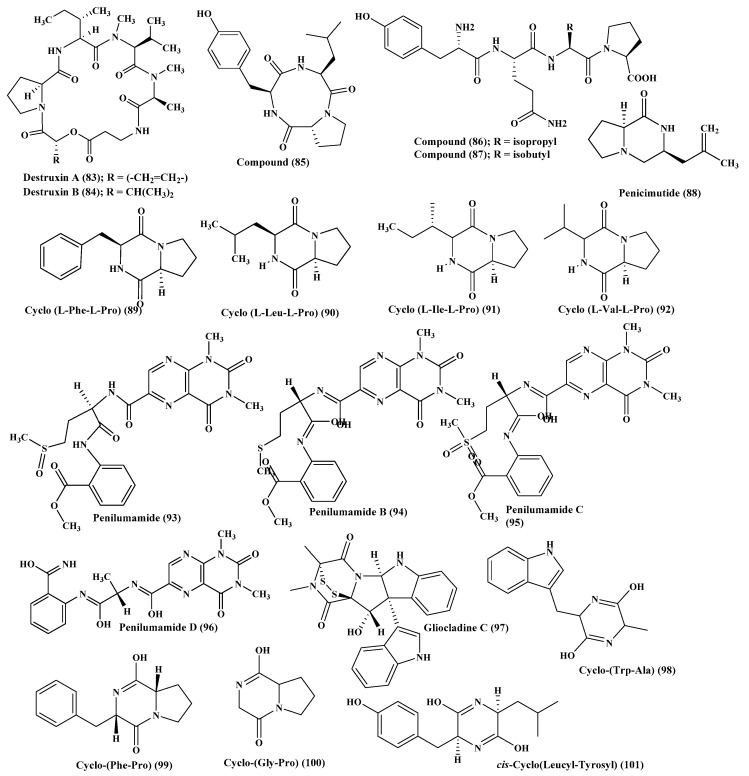
Peptides isolated from *Metarrhizium* and *Penicillium* species.

Destruxin cyclic depsipeptides, including A (**83**) and B (**84**), were reported to be produced by the sponge associated fungus, *Metarrhizium* sp., in addition to efrapeptins Eα (**4**), F (**6**), and G (**7**) [14] (Figure 5).

### 2.11. Penicillium

Many peptides were isolated from various *Penicillium* species, such as *Penicillium citrinum*, through the stimulation of silent genes using scandium chloride that resulted in the isolation of three new peptide derivatives (**85**–**87**). Compound (**85**) showed potent antibacterial activity against *S. aureus* with MIC value equals to 0.04 µM. However, compounds (**86–87**) displayed potent cytotoxic activity against MCF-7 and HCT115 cell lines showing IC_50_ equal to 38 and 37 nM, respectively, while compound (**85**) showed no activity [39]. Furthermore, penicimutide (**88**), which is a novel dipeptide in a cyclic form in addition to other previously reported dipeptides, namely cyclo (l-Phe-l-Pro) (**89**), cyclo (l-Leu-l-Pro) (**90**), cyclo (l-Ile-l-Pro) (**91**), and cyclo (l-Val-l-Pro) (**92**) were isolated from *P. purpurogenum*. Penicimutide **(88)** effectively inhibited growth of HeLa cells showing 39.4% as an inhibition rate at 100 µg/mL (0.5 µM) approaching that of the 5-fluorouracil that showed 41.4% at 100 µg/mL (0.77 µM) [40] (Figure 5).

Besides, penilumamide (**93**), as well as penilumamides B–D (**94**–**96**), were isolated from miscellaneous *Penicillium sp.* [41] in addition to gliocladine C (**97)**, cyclo-(Trp-Ala) (**98**), cyclo-(Phe-Pro) (**99**), and cyclo-(Gly-Pro) (**100**). Compound**s** (**97**–**100**) were assessed for their ability to inhibit HepG2 cells proliferation with different degrees in which gliocladine C (**97**) effectively inhibited HepG2 cells growth displaying IC_50_ value of 19.6 μM, whereas compounds (**98**–**100**) showed no relevant cytotoxic activity [42]. Moreover, *cis*-cyclo (Leucyl-Tyrosyl) (**101**) isolated from a sponge derived *Penicillium* species showed a significant inhibition to the biofilm formation estimated by 85% which consequently prevents bacterial growth that was further ascertained by a scanning electron microscope [43] (Figure 5).

### 2.12. Scytalidium 

**Figure 6:**
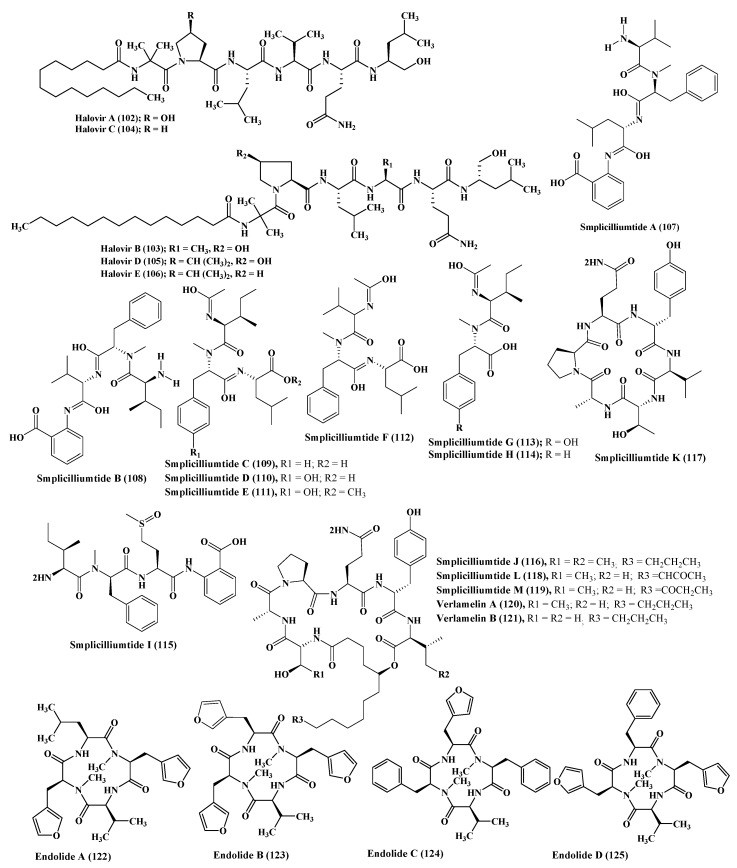
Peptides isolated from *Scytalidium*, *Simplicillium*, and *Stachylidium* species.

A marine fungus of the genus *Scytalidium* is highly popular for the production of linear, lipophilic peptides, termed the halovirs as halovirs A–E (**102**–**106**), which showed considerable in vitro antiviral activity against herpes simplex viruses of both type 1 and type 2. Further study on the structure activity relationship between the halovirs and their anti-viral activity showed that the presence of a *N*^α^-acyl chain, comprising of at least 14 carbons as well as the Aib-Pro dipeptide, are crucial to preserve the activity. Upon addition of Halovirs A (**102**), B (**103**), and C (**104**) to cells that are exposed to HSV-1 infection for 1 h, they displayed ED_50_ values of 1.1, 3.5, and 2.2 μM, respectively. Meanwhile, halovirs D (**105**) and E (**106**) showed ED_50_ values equal to 2.0 and 3.1 μM, respectively. Besides, halovir A (**102**) effectively prohibits HSV-1 replication showing ED50 value of 0.28 μM adopting the standard plaque reduction assay [44,45] (Figure 6).

### 2.13. Simplicillium 

A series of simplicilliumtide peptides were isolated from the marine fungus *Simplicillium obclavatum* namely; simplicilliumtides A–M (**107**–**119**) in addition to verlamelins A and B (**120**–**121**). They showed variable activities including antibacterial, antifungal, antiviral, antifouling, cytotoxic, and acetyl cholinesterase inhibitory activity. Simplicilliumtide D (**110**) displayed a potent antifouling effect versus the larvae of *Bugula neritina* with EC_50_ equal to 0.02 µM and LC_50_/EC_50_ equal to 100; however, simplicilliumtides A (**107**), E (**111**), G (**113**), and H (**114**) exhibited weak cytotoxic effect against human leukemia HL-60 and K562 cell lines with IC_50_ above 100 µM. In addition, simplicilliumtide J (**116**) exhibited potent antifungal activity versus *Curvularia australiensis* as well as *Aspergillus versicolor* in addition to a potent anti - HSV-1 displaying IC_50_ value of 14.0 µM that could be interpreted in virtue of the presence of lactone linkage and a fatty acid chain moiety [10,46] (Figure 6).

### 2.14. Stachylidium

Many N-methylated peptides have been isolated from *Stachylidium* sp., a fungus associated with marine sponge, which include endolides A–D (**122**–**125**) which showed a wide range of biological activities [47]. Endolide A (**122**) revealed potent binding activity to the vasopressin receptor 1A displaying a Ki of 7.04 µM, whereas endolide B (**123**) showed a pronounced binding to serotonin receptor 5HT2b evidenced by its Ki value, which is 0.77 µM [48] (Figure 6).

### 2.15. Talaromyces

**Figure 7:**
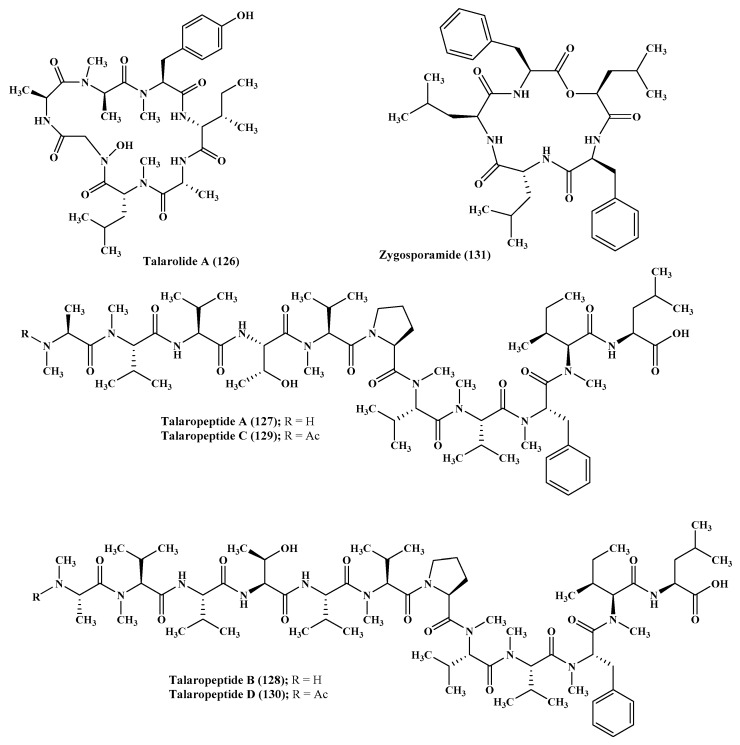
Peptides isolated from *Talaromyces* and *Zygosporium* species.

Talarolide A (**126**), in addition to a series of extensively N-methylated linear peptides named talaropeptides A–D (**127**–**130**), were isolated from *Talaromyces* sp., a fungus derived from a marine tunicate. Biological evaluation of the previously mentioned compounds revealed that compounds (**127**–**128**) only displayed pronounced antibacterial activity against the growth of *Bacillus subtilis*, a Gram positive bacterium, displaying IC_50_ values of 1.5 and 3.7 µM, respectively. Additionally compound talaropeptide A (**127)** showed a high stability to various rat proteases existing in plasma [49] (Figure 7).

### 2.16. Trichoderma

*Trichoderma* strains, derived from marine origin, afforded large amounts peptaibols, which are characterized by being small antimicrobial peptides (AMPs) that are expected to contribute to the antimicrobial defense of *Trichoderma. Trichoderma* strains produced 11- and 20-residue peptaibols; optimal yields with 1.4% and 2.3% of the fungal biomass for the 11- and 20-residue, respectively, were obtained on day 9 [50]. Generally, AMPs represent a large group of naturally occurring cationic and amphiphilic short peptides that act as first-line defense for many living organisms and are part of the innate immune system [51]. They act primarily via offering a protection to the host organisms versus the invasion of harmful organisms such as bacteria or fungi [52]. Their mode of action is completely different from traditional antibiotics as they modulate membrane stability and permeability. On the contrary, traditional antibiotics exert their effect mainly by interfering with bacterial metabolism, protein biosynthesis, or cell wall formation. Thus, low cross-resistance and an effective synergism could be achieved between AMPs and traditional antibiotics [53]. 

### 2.17. Zygosporium

*Zygosporium masonii*, a marine fungus that produced a cyclic depsipeptide termed zygosporamide (**131**) showed potent cytotoxic activity in the NCI’s 60 cell line panel with median GI_50_ equal to 9.1 μM. However, it revealed a notable selectivity versus the (Central Nervous System) CNS cancer cell SF-268 with GI_50_ equal to 6.5 nM, as well as to the renal cancer cell line RXF 393 showing GI_50_ less than 5.0 nM [54,55] (Figure 7).

## 3. Discussion and General Perspectives

In our analysis we found that 131 peptides from marine sources were isolated from 17 fungal strains. These peptides are either cyclic, composed of two amino acids (dipeptide), as in case of *Ascotricha* up to nine amino acids (nonapeptides) in *Emericella* or linear pentadecapeptide as presented in *Acremonium*. Many depsipeptides (from *Acremonium*, *Metarrhizium*, and *Zygosporium*), peptaibols in *Trichoderma* and N-methylated peptides from *Acremonium*, *Stachylidium*, and *Talaromyces* are also characterized. It is clearly noticed that genus *Aspergillus* was the most extensively studied and it might represent a rich source of peptides with promising biological activity. However, there is no evidence about the chemotaxonomic relation between the production of a certain class of peptides in a specified genus. That could be explained based on the fact that the number of the isolated compounds is not enough to make an in-depth study about this relation. Besides, peptides from marine sources possess strongly modulated structures either in its backbone or side chain comparable to peptides of plant or human origin, which undoubtedly is due to the harsh requirements of the environment in which they live. It worthy to mention that although most of these peptides contain many functional groups, such as carbonyl and amide groups, beside esters as in the case of depsipeptides which gave them the ability to interact with many molecular targets in the cells, most of these peptides are either inactive or showed weak activity. That could be explained by the fact that most of these peptides are weakly soluble in physiological fluids that limit most of their in vivo activity [56]. 

It was found that about 53% of the isolated peptides exhibited various biological activities represented mainly by cytotoxic, antimicrobial, and antiviral activity, while few of them showed antidiabetic, lipid lowering, and anti-inflammatory activity (Table 1). 

Regarding the cytotoxic effect of the previously mentioned peptides, about 35 bioactive peptides revealed cytotoxic activities against a panel of cancer cells. Twenty five peptides showed potent cytotoxic activity however the other ten peptides exerted weak activity. This was represented by cordyheptapeptides C (**1**) and E (**3**), which exerted notable activity on MCF-7, SF-268, and NCI-H460 cancer cells with IC_50_ ranging between 2.5 and 12.1 μM. Besides, pronounced cytotoxic potential was exerted by efrapeptins Eα (**4**), F (**6**), and G (**7**) against H125 cells with IC_50_ values of about 1.3 nM. Efrapeptin G (**7**) also showed potent cytotoxic activity versus murine cancer cells. Moreover, aspergillicins A–E (**17**–**21**) exerted substantial activity with LD_99_ values ranging between 0.03 and 0.07 μM. Additionally, effective stimulation to HT-29 cancer cells differentiation was exerted by cyclo-(l-Trp- l-Tyr) (**30**)**.** Furthermore, asperterrestide A (**38**) showed a potent cytotoxic effect versus U937 and MOLT4 human cancer; meanwhile, psychrophilin E (**50**), selectivity prohibited the proliferation of HCT116 (colon) cell line with an IC_50_ value of 28.5 mM. In addition, the cytotoxic effect of dictyonamide A (**70**) can be explained in virtue of its inhibitory effect on cyclin-dependent kinase 4 that showed IC_50_ value equals to 0.01 μM. Rostratins A–D (**77**–**80**) showed pronounced cytotoxic activity versus (HCT-116) the human colon carcinoma showing 20, 4.48, 1.6, and 34 nM as IC_50_ values, respectively. Moreover, microsporins A and B (**81**–**82**) exerted an effective cytotoxic effect versus human colon adenocarcinoma (HCT-116). Additionally, *Penicillium citrinum* represents a source of cytotoxic peptides in which compounds (**86**–**87**) exerted significant cytotoxic activity on MCF-7 and HCT115 cell lines showing IC_50_s equal to 38 and 37 nM, respectively. Penicimutide (**88**) also showed an effective inhibitory effect on HeLa cells displaying 39.4% as an inhibition rate at 100 µg/mL (0.01 μM) approaching that of the positive control 5-fluorouracil which showed 41.4% at 100 µg/mL. Furthermore, gliocladine C (**97**) effectively inhibited HepG2 cells growth displaying IC_50_ value of 19.6 μM whereas, zygosporamide (**131**) which showed potent cytotoxic activity. Weak cytotoxic activities were exerted by sclerotide A (**31**), sclerotide B (**32**), cotteslosins A (**57**), and B (**58**), in addition to similanamide (**33**) which displayed weak cytotoxic activity versus MCF-7, A373, and NCI-H460 cancer cells. In addition, aspersymmetide A (**55**) showed weak cytotoxicity versus NCI-H292 as well as A431 cell lines at 10 µM; meanwhile, simplicilliumtide A (**107**), E (**111**), G (**113**), and H (**114**) exhibited weak cytotoxic effect versus either human leukemia HL-60 or K562 cell line.

Concerning the antimicrobial and antiviral activities, 23 peptides exerted pronounced activity, among which nine revealed a potent inhibitory effect on bacterial growth, three showed pronounced antifungal activity, whereas eleven exhibited promising antiviral activity. Pronounced antibacterial activity was observed for RHM (**8**) and efrapeptin G (**6**) against *Staphylococcus epidermidis* with MIC values equal to 0.015 and 0.049 μM, respectively. In addition, lajollamide A (**69**) showed a mild antimicrobial effect against Gram-positive bacteria exerting 61% and 30% inhibition in bacterial growth for *Bacillus subtilis* and *Staphylococcus epidermidis* at 100 μM. Besides, unguisin A (**42**) and emericellamide B (**76**) showed considerable antibacterial activity against MRSA (methicillin resistant *Staphylococcus aureus* strains) with MIC values equal to 3.8 and 6.0 µM, respectively, whereas compound (**85**) isolated from *Penicillium citrinum* also showed pronounced antibacterial activity against *S. aureus* displaying 0.04 µM as MIC. However, the effective antimicrobial activity of *cis*-cyclo (Leucyl-Tyrosyl) (**101**) can be explained in virtue of its effective inhibitory effect on bacterial biofilm formation, which is estimated by 85%. Additionally, talaropeptides A–B (**127**–**128**), showed notable antibacterial activity against the growth of *Bacillus subtilis*, a Gram positive bacterium, displaying IC_50_ values of 1.5 and 3.7 µM, respectively. Noteworthy to highlight is that *Trichoderma* strains afforded large amounts of peptaibols which are characterized by being small antimicrobial peptides (AMPs) that are expected to contribute to the antimicrobial defense of *Trichoderma.* For the antifungal activity, sclerotide A (**31**), sclerotide B (**32**), and simplicilliumtide J (**116**) showed notable antifungal activity. 

A considerable number of fungal peptides showed powerful antiviral activity exemplified by asperterrestide A (**38**) which exerted potent potential antiviral activity versus an influenza virus of H1N1 as well as H3N2 strains that could be relied upon the presence of a 3-OH-N-CH3-Phe moiety which is rare in nature. Moreover, aspergillipeptides D–G (**62**–**65**), showed a pronounced antiviral effect versus herpes simplex virus type 1 (HSV-1) displaying IC_50_ of 9.5 and 19.8 µM, respectively. Halovirs A–E (**102**–**106**) showed considerable in vitro antiviral activity against herpes simplex viruses of both type 1 and type 2 which is ultimately attributed to the presence of a *N*^α^-acyl chain, comprising of at least 14 carbons as well as the Aib-Pro dipeptide. However, simplicilliumtide J (**116**) exerted a potent anti - HSV-1 displaying IC_50_ value of 14.0 µM which could be interpreted in virtue of the presence of lactone linkage and a fatty acid chain moiety.

The anti-inflammatory activity of some of the isolated peptides can be explained via exerting different mechanisms where compounds (**60**–**61**) obtained from *Aspergillus* at 10 µM effectively inhibited IL-10 expression of the LPS-induced THP-1 cells showing 84.3% and 78.1% inhibition, respectively. Besides, 14-hydroxy-cyclopeptine (**66**) effectively inhibits nitric oxide production displaying IC_50_ of 0.14 μM in recombinant mouse interferon-γ -activated macrophage-like cell line. 

Antidiabetic and lipid lowering activity was also observed for some of the isolated fungal peptides, such as terrelumamides A (**34**) and B (**35**), which displayed a promising improvement in insulin sensitivity as determined by the utilization of mesenchymal cells of a bone marrow origin obtained from human adopting an adipogenesis model. Additionally, psychrophilin G (**52**) exhibited pronounced lipid-reducing activity.

Additionally, miscellaneous activities were reported for the isolated bioactive peptides in which Simplicilliumtide D (**110**) displayed a potent antifouling effect versus the larvae of *Bugula neritina*. Moreover, endolide A (**122**) revealed potent binding activity to the vasopressin receptor 1A displaying a Ki of 7.04 µM whereas endolide B (**123**) showed a pronounced binding to serotonin receptor 5HT2b evidenced by its Ki value which is 0.77 µM. Talaropeptide A (**126**) showed a high stability to various rat proteases existing in plasma, however, microsporins A and B (**81**–**82**) effectively inhibit histone deacetylase.

**Figure 8:**
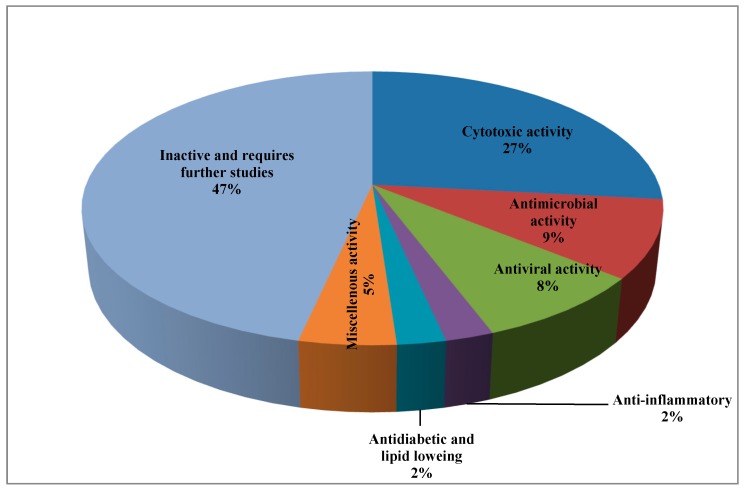
Percentages of isolated peptides with respect to their biological activity represented by a pie chart.

Noteworthy to mention is that about 47% of the isolated peptides showed no activity with respect to the examined biological activity and thus required further in depth biological assessment. A pie chart summarizing the percentages of isolated peptides with respect to their biological activity is represented in Figure 8. 

## 4. Conclusions

Herein, we concluded that about 131 peptides were isolated from marine sources from seventeen fungal strains. Some of them exhibited much biological activity represented mainly by cytotoxic, antimicrobial, and antiviral activity, meanwhile, few of them showed antidiabetic, lipid lowering, and anti-inflammatory activity; however, others should be deeply assessed for their biological potential. Thus, if active natural products are needed for drug development, further investigations of marine fungi are recommended. In addition, it is a challenge to explore more peptides from fungal origin and so it is recommended to confirm their biological activities.

## Figures and Tables

**Table 1:** Bioactive peptides isolated from marine associated fungi, their sources, and biological activities.

Compound	*Genus*	Biological Activity	Reference
Cordyheptapeptide C (**1**)	*Acremonium*	Cytotoxic activity versus MCF-7, SF-268 and NCI-H460 cancer cells	[12]
Cordyheptapeptide E (**3**)	*Acremonium*	Cytotoxic activity versus MCF-7, SF-268 and NCI-H460 cancer cells	[12]
Efrapeptin Eα (**4**)	*Acremonium*	Cytotoxic activity versus H125 cells	[13,14]
Efrapeptin F (**6**)	*Acremonium*	Cytotoxic activity versus H125 cells	[13,14]
Efrapeptin G (**7**)	*Acremonium*	Cytotoxic activity versus H125 and HCT-116 cells Antibacterial activity versus *Staphylococcus epidermidis*	[13,14]
RHM1 (**8**)	*Acremonium*	Antibacterial activity versus *Staphylococcus epidermidis*	[13,14]
Aspergillicins A–E (**17**–**21**)	*Aspergillus*	Cytotoxic activity	[11]
Cyclo-(L-Trp-L-Tyr) (**30**)	*Aspergillus*	Cytotoxic activity versus HT-29 cancer cells	[17]
Sclerotide A (**31**)	*Aspergillus*	Antifungal activity versus *Candida albicans*	[18]
Sclerotide B (**32**)	*Aspergillus*	Antifungal activity versus *Candida albicans* Antibacterial activity versus *Pseudomonas aeruginosa* Weak cytotoxic activity versus HL-60 cell ( IC_50_ > 50 µM)	[18]
Similanamide (**33**)	*Aspergillus*	Weak cytotoxic activity versus MCF-7, A373 and NCI-H460 cancer cells (GI_50_ > 0.15 µM)	[19]
Terrelumamide A (**34**)	*Aspergillus*	Improve insulin sensitivity	[20,21,22]
Terrelumamide B (**35**)	*Aspergillus*	Improve insulin sensitivity	[20,21,22]
Compound (**38**)	*Aspergillus*	Cytotoxic activity versus U937 and MOLT4 human cancer cell lines Antiviral activity versus influenza virus of H1N1 and H3N2 strains	[20,21,22].
Psychrophilin E (**50**)	*Aspergillus*	Cytotoxic activity versus HCT116	[29]
Psychrophilin G (**52**)	*Aspergillus*	Lipid-lowering activity	[25]
Aspersymmetide A (**55**)	*Aspergillus*	Weak cytotoxic activity versus NCI-H292 as well as A431 cell lines ( IC_50_ > 10 µM)	[26]
Cotteslosin A (**57**)	*Aspergillus*	Weak cytotoxic activity versus human melanoma (MM418c5), prostate (DU145) and breast (T47D) cells ( EC_50_> 0.1 µM	[27]
Diketopiperazine dimer (**60**)	*Aspergillus*	Anti-inflammatory activity against IL-10 expression of the LPS-induced THP-1 cells	[28]
Cyclic tetrapeptide (**61**)	*Aspergillus*	Anti-inflammatory activity against IL-10 expression of the LPS-induced THP-1 cells	[28]
Aspergillipeptid D (**62**)	*Aspergillus*	Antiviral activity versus herpes simplex virus type 1 (HSV-1) and acyclovir resistant clinical isolates	[30]
Aspergillipeptide E (**63**)	*Aspergillus*	Antiviral activity versus herpes simplex virus type 1 (HSV-1)	[30]
14-Hydroxy-cyclopeptine (**66**)	*Aspergillus*	Inhibition of nitric oxide production	[31]
Lajollamide A (**69**)	*Asteromyces*	Weak antimicrobial activity versus Bacillus subtilis and Staphylococcus epidermidis. (MIC > 50 µM)	[33]
Dictyonamide A (**70**)	*Ceratodictyon*	Inhibition on cyclin-dependent kinase 4	[34]
Clonostachysin A (**72**)	*Clonostachys*	Inhibition of dinoflagellate *Prorocentrum micans*	[35]
Clonostachysin B (**73**)	*Clonostachys*	Inhibition of dinoflagellate *Prorocentrum micans*	[35]
Unguisin A (**42**)	*Emericella*	Antimicrobial activity versus MRSA (methicillin resistant *Staphylococcus aureus* strains)	[37]
Emericellamide B (**76**)	*Emericella*	Antimicrobial activity versus MRSA (methicillin resistant *Staphylococcus aureus* strains)	[37]
Rostratins A–D (**77**–**80**)	*Exserohilum*	Cytotoxic activity versus (HCT-116) the human colon carcinoma	[38]
Microsporin A (**81**)	*Microsporum*	Inhibition of histone deacetylase Cytotoxic activity versus human colon adenocarcinoma (HCT-116) and the 60 cancer cell panel of the National Cancer Institute	[39]
Microsporin B (**82**)	*Microsporum*	Inhibition of histone deacetylase Cytotoxic activity versus human colon adenocarcinoma (HCT-116)	[39]
Compound (**85**)	*Penicillium*	Antibacterial activity versus *S. aureus*	[39]
Compounds (**86**–**87**)	*Penicillium*	Cytotoxic activity versus MCF-7 and HCT115 cell lines	[39]
Penicimutide (**88**)	*Penicillium*	Cytotoxic activity versus HeLa cells	[40]
Gliocladine C (**97**)	*Penicillium*	Cytotoxic activity versus HepG2 cells	[42]
*cis*-Cyclo (Leucyl-Tyrosyl) (**101**)	*Penicillium*	Inhibition to the biofilm formation	[43]
Halovir A (**102**)	*Scytalidium*	Antiviral activity versus herpes simplex viruses of both type 1 and type 2 and HSV-1	[44,45]
Halovirs B–E (**103**–**106**)	*Scytalidium*	Antiviral activity versus herpes simplex viruses of both type 1 and type 2	[44,45]
Simplicilliumtide A (**107**)	*Simplicillium*	Weak cytotoxic activity versus human leukemia HL-60 and K562 cell line	[10,46]
Simplicilliumtide D (**110**)	*Simplicillium*	Antifouling effect versus the larvae of *Bugula neritina*	[10,46]
Simplicilliumtides E (**111**), G (**113**), and H (**114**)	*Simplicillium*	Weak cytotoxic effect versus human leukemia HL-60 and K562 cell line ( IC_50_ > 100 µM)	[10,46]
Simplicilliumtide J (**116**)	*Simplicillium*	Antifungal activity versus Curvularia australiensis and Aspergillus versicolor Antiviral activity versus HSV-1	[10,46]
Endolide A (**122**)	*Stachylidium*	Binding activity to the vasopressin receptor 1A	[48]
Endolide B (**123**)	*Stachylidium*	Binding to serotonin receptor 5HT2b	[48]
Talaropeptide A (**127**)	*Talaromyces*	Antibacterial activity versus *Bacillus subtilis*	[49]
Talaropeptide B (**128**)	*Talaromyces*	Antibacterial activity versus *Bacillus subtilis*	[49]
Zygosporamide (**131**)	*Zygosporium*	Cytotoxic activity versus NCI’s 60 cell line panel, CNS cancer cell line SF-268, renal cancer cell line RXF 393	[54,55]

## References

[1] Jin L., Quan C., Hou X., Fan S. (2016). Potential pharmacological resources: Natural bioactive compounds from marine-derived fungi. Mar. Drugs.

[2] Rateb M.E., Ebel R. (2011). Secondary metabolites of fungi from marine habitats. Nat. Prod. Rep..

[3] Schueffler A., Anke T. (2014). Fungal natural products in research and development. Nat. Prod. Rep..

[4] Blunt J.W., Copp B.R., Keyzers R.A., Munro M.H., Prinsep M.R. (2015). Marine natural products. Nat. Prod. Rep..

[5] Blunt J.W., Copp B.R., Keyzers R.A., Munro M.H., Prinsep M.R. (2017). Marine natural products. Nat. Prod. Rep..

[6] Kang H.K., Lee H.H., Seo C.H., Park Y. (2019). Antimicrobial and immunomodulatory properties and applications of marine-derived proteins and peptides. Mar. Drugs.

[7] Saleem M., Ali M.S., Hussain S., Jabbar A., Ashraf M., Lee Y.S. (2007). Marine natural products of fungal origin. Nat. Prod. Rep..

[8] Sable R., Parajuli P., Jois S. (2017). Peptides, peptidomimetics, and polypeptides from marine sources: A wealth of natural sources for pharmaceutical applications. Mar. Drugs.

[9] Abdel-Wahab N.M., Harwoko H., Müller W.E., Hamacher A., Kassack M.U., Fouad M.A., Kamel M.S., Lin W., Ebrahim W., Liu Z. (2019). Cyclic heptapeptides from the soil-derived fungus Clonostachys rosea. Bioorg. Med. Chem..

[10] Liang X., Nong X.-H., Huang Z.-H., Qi S.-H. (2017). Antifungal and antiviral cyclic peptides from the deep-sea-derived fungus *Simplicillium obclavatum* EIODSF 020. J. Agric. Food Chem..

[11] Capon R.J., Skene C., Stewart M., Ford J., Richard A., Williams L., Lacey E., Gill J.H., Heiland K., Friedel T. (2003). Aspergillicins A–E: Five novel depsipeptides from the marine-derived fungus *Aspergillus carneus*. Org. Biomol. Chem..

[12] Chen Z., Song Y., Chen Y., Huang H., Zhang W., Ju J. (2012). Cyclic heptapeptides, cordyheptapeptides C–E, from the marine-derived fungus *Acremonium persicinum* SCSIO 115 and their cytotoxic activities. J. Nat. Prod..

[13] Boot C.M., Tenney K., Valeriote F.A., Crews P. (2006). Highly N-methylated linear peptides produced by an atypical sponge-derived *Acremonium* sp.. J. Nat. Prod..

[14] Boot C.M., Amagata T., Tenney K., Compton J.E., Pietraszkiewicz H., Valeriote F.A., Crews P. (2007). Four classes of structurally unusual peptides from two marine-derived fungi: Structures and bioactivities. Tetrahedron..

[15] Xie L.-R., Li D.-Y., Wang P.-L., Hua H.-M., Wu X., Li Z.-L. (2013). A new 3, 4-seco-lanostane triterpenoid from a marine-derived fungus *Ascotricha* sp. ZJ-M-5. Acta Pharm. Sin..

[16] Uchoa P.K.S., Pimenta A.T., Braz-Filho R., de Oliveira M.d.C.F., Saraiva N.N., Rodrigues B.S., Pfenning L.H., Abreu L.M., Wilke D.V., Florêncio K.G. (2017). New cytotoxic furan from the marine sediment-derived fungi *Aspergillus niger*. Nat. Prod. Rep..

[17] Zhang Y., Li X.-M., Feng Y., Wang B.-G. (2010). Phenethyl-α-pyrone derivatives and cyclodipeptides from a marine algous endophytic fungus *Aspergillus niger* EN–13. Nat. Prod. Rep..

[18] Zheng J., Zhu H., Hong K., Wang Y., Liu P., Wang X., Peng X., Zhu W. (2009). Novel cyclic hexapeptides from marine-derived fungus, *Aspergillus sclerotiorum* PT06-1. Org. Lett..

[19] Prompanya C., Fernandes C., Cravo S., Pinto M., Dethoup T., Silva A., Kijjoa A. (2015). A new cyclic hexapeptide and a new isocoumarin derivative from the marine sponge-associated fungus *Aspergillus similanensis* KUFA 0013. Mar. Drugs.

[20] You M., Liao L., Hong S., Park W., Kwon D., Lee J., Noh M., Oh D.-C., Oh K.-B., Shin J. (2015). Lumazine peptides from the marine-derived fungus *Aspergillus terreus*. Mar. Drugs.

[21] Luo X.-W., Lin Y., Lu Y.-J., Zhou X.-F., Liu Y.-H. (2019). Peptides and polyketides isolated from the marine sponge-derived fungus *Aspergillus terreus* SCSIO 41008. Chin. J. Nat. Med..

[22] He F., Bao J., Zhang X.-Y., Tu Z.-C., Shi Y.-M., Qi S.-H. (2013). Asperterrestide A, a cytotoxic cyclic tetrapeptide from the marine-derived fungus *Aspergillus terreus* SCSGAF0162. J. Nat. Prod..

[23] Yang W.-C., Bao H.-Y., Liu Y.-Y., Nie Y.-Y., Yang J.-M., Hong P.-Z., Zhang Y. (2018). Depsidone Derivatives and a cyclopeptide produced by marine fungus *Aspergillus unguis* under chemical induction and by its plasma induced mutant. Molecules.

[24] Capon R.J., Ratnayake R., Stewart M., Lacey E., Tennant S., Gill J.H. (2005). Aspergillazines A–E: Novel heterocyclic dipeptides from an Australian strain of *Aspergillus unilateralis*. Org. Biomol. Chem..

[25] Peng J., Gao H., Zhang X., Wang S., Wu C., Gu Q., Guo P., Zhu T., Li D. (2014). Psychrophilins E–H and versicotide C, cyclic peptides from the marine-derived fungus *Aspergillus versicolor* ZLN-60. J. Nat. Prod..

[26] Hou X.-M., Zhang Y.-H., Hai Y., Zheng J.-Y., Gu Y.-C., Wang C.-Y., Shao C.-L. (2017). Aspersymmetide A, a new centrosymmetric cyclohexapeptide from the marine-derived fungus *Aspergillus versicolor*. Mar. Drugs.

[27] Fremlin L.J., Piggott A.M., Lacey E., Capon R.J. (2009). Cottoquinazoline A and cotteslosins A and B, metabolites from an Australian marine-derived strain of *Aspergillus versicolor*. J. Nat. Prod..

[28] Liu J., Gu B., Yang L., Yang F., Lin H. (2018). New anti-inflammatory cyclopeptides from a sponge-derived fungus *Aspergillus violaceofuscus*. Front. Chem..

[29] Ebada S.S., Fischer T., Hamacher A., Du F.-Y., Roth Y.O., Kassack M.U., Wang B.-G., Roth E.H. (2014). Psychrophilin E, a new cyclotripeptide, from co-fermentation of two marine alga-derived fungi of the genus Aspergillus. Nat. Prod. Res..

[30] Ma X., Nong X.-H., Ren Z., Wang J., Liang X., Wang L., Qi S.-H. (2017). Antiviral peptides from marine gorgonian-derived fungus Aspergillus sp. SCSIO 41501. Tetrahedron Lett..

[31] Zhou X., Fang P., Tang J., Wu Z., Li X., Li S., Wang Y., Liu G., He Z., Gou D. (2016). A novel cyclic dipeptide from deep marine-derived fungus Aspergillus sp. SCSIOW2. Nat. Prod. Res..

[32] Zheng C.J., Wu L.Y., Li X.B., Song X.M., Niu Z.G., Song X.P., Chen G.Y., Wang C.Y. (2015). Structure and Absolute Configuration of Aspergilumamide A, a novel lumazine peptide from the mangrove-derived fungus *Aspergillus* sp.. Helv. Chim. Acta..

[33] Gulder T., Hong H., Correa J., Egereva E., Wiese J., Imhoff J., Gross H. (2012). Isolation, structure elucidation and total synthesis of lajollamide A from the marine fungus *Asteromyces cruciatus*. Mar. Drugs.

[34] Komatsu K., Shigemori H., Kobayashi J.i. (2001). Dictyonamides A and B, new peptides from marine-derived fungus. J. Org. Chem..

[35] Adachi K., Kanoh K., Wisespongp P., Nishijima M., Shizuri Y. (2005). Clonostachysins A and B, new anti-dinoflagellate cyclic peptides from a marine-derived fungus. J. Antibiot..

[36] Malmstrøm J. (1999). Unguisins A and B: New cyclic peptides from the marine-derived fungus *Emericella unguis*. J. Nat. Prod..

[37] Oh D.-C., Kauffman C.A., Jensen P.R., Fenical W. (2007). Induced production of emericellamides A and B from the marine-derived fungus *Emericella* sp. in competing co-culture. J. Nat. Prod..

[38] Tan R.X., Jensen P.R., Williams P.G., Fenical W. (2004). Isolation and structure assignments of rostratins A− D, cytotoxic disulfides produced by the marine-derived fungus *Exserohilum r ostratum*. J. Nat. Prod..

[39] Gu W., Cueto M., Jensen P.R., Fenical W., Silverman R.B. (2007). Microsporins A and B: New histone deacetylase inhibitors from the marine-derived fungus *Microsporum* cf. *gypseum* and the solid-phase synthesis of microsporin A. Tetrahedron.

[40] Wang N., Cui C.-B., Li C.-W. (2016). A new cyclic dipeptide penicimutide: The activated production of cyclic dipeptides by introduction of neomycin-resistance in the marine-derived fungus *Penicillium purpurogenum* G59. Arch. Pharm. Res..

[41] Meyer S.W., Mordhorst T.F., Lee C., Jensen P.R., Fenical W., Köck M. (2010). Penilumamide, a novel lumazine peptide isolated from the marine-derived fungus, *Penicillium* sp. CNL-338. Org. Biomol. Chem..

[42] Hong R., Yang Y. (2011). Antitumor metabolites from marine sediment derived *Penicillium* sp. WF-06. World Notes Antibiots.

[43] Scopel M., Abraham W.-R., Henriques A.T., Macedo A.J. (2013). Dipeptide cis-cyclo (Leucyl-Tyrosyl) produced by sponge associated Penicillium sp. F37 inhibits biofilm formation of the pathogenic Staphylococcus epidermidis. Bioorg. Med. Chem. Lett..

[44] Rowley D.C., Kelly S., Jensen P., Fenical W. (2004). Synthesis and structure–activity relationships of the halovirs, antiviral natural products from a marine-derived fungus. Bioorg. Med. Chem..

[45] Rowley D.C., Kelly S., Kauffman C.A., Jensen P.R., Fenical W. (2003). Halovirs A–E, new antiviral agents from a marine-derived fungus of the genus *Scytalidium*. Bioorg. Med. Chem..

[46] Liang X., Zhang X.-Y., Nong X.-H., Wang J., Huang Z.-H., Qi S.-H. (2016). Eight linear peptides from the deep-sea-derived fungus *Simplicillium obclavatum* EIODSF 020. Tetrahedron..

[47] El Maddah F., Kehraus S., Nazir M., Almeida C., König G.M. (2016). Insights into the biosynthetic origin of 3-(3-furyl) alanine in *Stachylidium* sp. 293 K04 tetrapeptides. J. Nat. Prod..

[48] Almeida C., Maddah F.E., Kehraus S., Schnakenburg G., König G.M. (2016). Endolides A and B, vasopressin and serotonin-receptor interacting N-methylated peptides from the sponge-derived fungus *Stachylidium* sp.. Organic letters..

[49] Dewapriya P., Khalil Z.G., Prasad P., Salim A.A., Cruz-Morales P., Marcellin E., Capon R.J. (2018). Talaropeptides AD: Structure and biosynthesis of extensively N-methylated linear peptides from an Australian marine tunicate-derived *Talaromyces* sp.. Front. Chem..

[50] Van Bohemen A.-I., Zalouk-Vergnoux A., Poirier L., Phuong N.N., Inguimbert N., Salah K.B.H., Ruiz N., Pouchus Y.F. (2016). Development and validation of LC–MS methods for peptaibol quantification in fungal extracts according to their lengths. J. Chromatogr. B.

[51] Herbel V., Wink M. (2016). Mode of action and membrane specificity of the antimicrobial peptide snakin-2. PeerJ.

[52] Herbel V., Schäfer H., Wink M. (2015). Recombinant production of snakin-2 (an antimicrobial peptide from tomato) in *E. coli* and analysis of its bioactivity. Molecules.

[53] Fan X., Schäfer H., Reichling J., Wink M. (2013). Bactericidal properties of the antimicrobial peptide Ib-AMP4 from *Impatiens balsamina* produced as a recombinant fusion-protein in *Escherichia coli*. Biotechnol. J..

[54] Torres-García C., Pulido D., Albericio F., Royo M., Nicolás E. (2014). Triazene as a powerful tool for solid-phase derivatization of phenylalanine containing peptides: *Zygosporamide* analogues as a proof of concept. J. Org. Chem..

[55] Oh D.-C., Jensen P.R., Fenical W. (2006). Zygosporamide, a cytotoxic cyclic depsipeptide from the marine-derived fungus Zygosporium masonii. Tetrahedron Lett..

[56] Fosgerau K., Hoffmann T. (2015). Peptide therapeutics: Current status and future directions. Drug Discov. Today..

